# Optimization, Validation, and Application of Cleanup-Coupled Liquid Chromatography–Tandem Mass Spectrometry for the Simultaneous Analyses of 35 Mycotoxins and Their Derivatives in Cereals

**DOI:** 10.3390/foods13223617

**Published:** 2024-11-13

**Authors:** Dan-Bi Kim, Miso Nam, Yong-Suk Kim, Min-Sun Kim

**Affiliations:** 1 Food Analysis Research Center, Korea Food Research Institute, Wanju 55365, Republic of Korea; dbkim1022@kfri.re.kr (D.-B.K.); msnam@kfri.re.kr (M.N.); 2 Department of Food Science and Technology, Jeonbuk National University, Jeonju 54896, Republic of Korea

**Keywords:** mycotoxin, ergot alkaloids, cleanup, method validation, simultaneous determination, co-occurrence

## Abstract

Mycotoxins occur singly or as co-contaminants and are primarily present in carbohydrate-rich foods such as cereals and cereal-based products. To effectively monitor mycotoxin co-contamination in cereals and cereal-based products, the simultaneous analysis of mycotoxins and their derivatives is required. Therefore, we coupled cleanup with LC-MS/MS for the rapid and robust quantitation of 35 analytes in wheat samples, including ergot alkaloids (EAs), which are rarely included in such analyses. To investigate the effects of different mycotoxin types on adsorbents, various dispersive solid-phase extraction sorbents were evaluated; a C_18_ end-capped sorbent exhibited the most effective cleanup performance. The method was validated by analyzing samples fortified with the mycotoxins at three concentration levels. The results exhibited high linearity, high recoveries, and repeatability. The methodology was applied for commercial cereal samples. The cereal samples were found to be 74% contaminated, and two samples measured levels of EAs at 609.63 μg/kg and 294.93 μg/kg, exceeding the limits defined by the EU for rye milling products. These findings highlight the validity of our novel method and the necessity of continuously monitoring mycotoxin levels in cereals to ensure food safety.

## 1. Introduction

Mycotoxins are vital indicators of food safety; their detection and analysis are crucial for the preemptive management of the safety concerns associated with the consumption of cereals and cereal-based products. The mycotoxins found in food that are associated with the highest potential risk to human and animal health are aflatoxins B1, B2, G1, and G2 (AFB1, AFB2, AFG1, and AFG2); ochratoxins A and B (OTA and OTB); trichothecenes, such as deoxynivalenol (DON), nivalenol (NIV), diacetoxyscirpenol (DAS), T-2 toxin (T-2), and HT-2 toxin (HT-2); fumonisins B1 and B2 (FB1 and FB2); zearalenone (ZEN); and ergot alkaloids (EAs) [1,2]. These mycotoxins are associated with various human diseases, including toxic hepatitis, hemorrhage, edema, immunosuppression, hepatic carcinoma, equine leukoencephalomalacia, esophageal cancer, and kidney failure [3]. For instance, AFB1 is classified as a Class I human carcinogen, and FB1 and OTA are classified as Class 2 B (probable human) carcinogens by the International Agency for Research on Cancer (IARC) [4].

Mycotoxin contamination usually involves the concurrent presence of low doses of multiple mycotoxins rather than high doses of a single mycotoxin; combinations of specific toxins have been reported to have adverse effects on animals and humans at the cellular level [5,6]. Several studies have reported the co-existence of mycotoxins in cereals and cereal-based products [7,8]. Multiple methods have been developed and validated for the simultaneous analysis of multiple mycotoxins with high sensitivity and accuracy [9]. However, only a few studies have reported the simultaneous analysis of EAs, along with other mycotoxins, in cereals [10,11,12]. EAs are classified as tryptophan-derived alkaloids, distinct from other mycotoxins. Notably, all EAs have a characteristic tetracyclic ergoline ring structure. EAs consist of ergometrine (Em), ergosine (Es), ergotamine (Et), ergocornine (Eco), ergocryptine (Ecry), ergocristine (Ecri), and the diastereoisomers ergometrinine (Emn), ergosinine (Esn), ergotaminine (Etn), ergocorninine (Econ), ergokriptinine (Ecryn), and ergocristinine (Ecrin) centered around the C_8_ position. EAs were considered significantly toxic for the European Union to establish maximum levels (MLs) for their presence in cereals and cereal products (100–500 μg/kg) in 2021 in order to ensure food safety; the MLs have since been further decreased to the range of 50–250 μg/kg in January 2024. Other major countries, such as Australia (500 mg/kg), Canada (0.1 mg/kg), New Zealand (100 μg/kg), and China (0.01% of the total EA contents in grains), have established and managed MLs for EAs in cereals and cereal products [13]. As regulations on EAs have increasingly become stringent across many regions, there is a growing demand for information on the levels and distributions of the mycotoxins in cereals, even at concentrations well below the legal limits. Consequently, it is necessary to develop simultaneous analysis methods for various mycotoxins and investigate their contamination levels and distributions continuously to ensure the safety of cereals and cereal-based products.

Over the last decade, the widespread utilization of highly sensitive and selective UHLC-MS/MS instrumentation has been observed for the analysis of multiple mycotoxins in food, in combination with sample preparation methods, including SPE, the QuEChERS (Quick, Easy, Cheap, Effective, Rugged, and Safe) method, and immunoaffinity columns [14,15,16]. The SPE method involves multiple steps, such as cartridge conditioning and solvent passage, making it complex and time-consuming, and requires the use of large volumes of organic solvents, which is environmentally undesirable. In the case of immunoaffinity columns, they are selectively capable of separating only specific groups, such as aflatoxins, ochratoxins, and EAs, making them unsuitable for simultaneous mycotoxin analysis. As for d-SPE, it is used extensively in the cleanup step in the QuEChERS method, where the sample and sorbent are mixed directly for purification, simplifying the sample preparation process compared to traditional SPE and leading to a larger contact area between the sample and sorbent, resulting in higher purification efficiency [17].

The objective of the present study was to develop a convenient, rapid, and specific cleanup-coupled ultra-high-performance liquid chromatography–tandem mass spectroscopy (UPLC-MS/MS) method for the simultaneous analysis of multiple mycotoxins, including EAs, and their derivatives in cereals. The specificity, linearity, range, accuracy, precision, limit of detection (LOD), and limit of quantitation (LOQ) were based on European Commission Decision 2002/657/EC, and measurement uncertainty was also evaluated. In addition, a validated analytical method was used to investigate the presence of mycotoxins in 23 cereal products marketed in South Korea.

## 2. Materials and Methods

### 2.1. Chemicals

Standards of AFB1, AFB2, AFG1, AFG2, Fumonisin B3 (FB3), Em, Es, Et, Eco, Ecry, Ecri, and diastereoisomers Emn, Esn, Etn, Econ, Ecryn, and Ecrin were purchased from Romer Labs (Tulln, Austria). OTA, OTB, FB1, FB2, T-2, HT-2, ZEA, NIV, DON, DAS, fusarenone X (Fus-X), 3-acetyl deoxynivalenol (3-AcDON), and 15-acetyl deoxynivalenol (15-AcDON) were obtained from Sigma-Aldrich (St. Louis, MO, USA). Ochratoxin C (OTC) and 15-acetoxyscirpenol (15-AS) were purchased from Cayman Chemical Company (Ann Arbor, MI, USA), and zearalenone 4-sulfate (ZEA-4S) from TRC (Toronto, ON, Canada).

The sorbents tested for dispersive solid-phase extraction (d-SPE), including LC-Florisil SPE (Florisil), Supel QuE (Z-sep), silica gel (average particle size, 35–60 mesh), aluminum oxide (Al_2_O_3_), and formic acid, were obtained from Sigma-Aldrich (St. Louis, MO, USA). The primary secondary amine (PSA) sorbent (average particle size, 40–120 μm) and C_18_ end-capped (C_18_ EC) sorbent (average particle size, 40–120 μm) were purchased from Agilent Technologies (Lake Forest, CA, USA). All solvents used were of LC-MS grade and purchased from Fisher Scientific (Pittsburgh, PA, USA).

### 2.2. Standard Solution Preparation

Standard stock solutions were prepared in acetonitrile for all the mycotoxins except the FBs, which were dissolved in a water/acetonitrile solution (50:50, *v*/*v*). The standard stock solutions were stored at −20 °C. The working standard solutions were obtained by diluting each stock solution with a water/methanol solution (50:50, *v*/*v*) to concentrations of 5–100 μg/kg.

### 2.3. Blank Matrix and Sample Collection

A blank sample (non-contaminated whole-wheat flour) (Romer Labs Inc., Tulln, Austria) was used for quality control. A total of 23 cereal samples were collected from the local market; information regarding the raw material, origin, and expiration date is provided in Table S1 of the Supplementary Material. All samples were immediately dispensed into polyethylene bags and stored at −20 °C until extraction.

### 2.4. UPLC-MS/MS Analysis

The quantification of mycotoxins and their derivatives was performed using an Agilent 1200 HPLC system (Agilent Technologies) and a 4500 QTRAP mass spectrometer (AB SCIEX, Darmstadt, Germany) coupled to an electrospray ionization (ESI) source in the positive/negative-switching scan mode and multiple-reaction monitoring (MRM) mode. The other parameters for mass detection were the spray voltage: +5500 V (positive) and −4500 V (negative); entrance potential: +10 V (positive) and −10 V (negative); source temperature: 550 °C; curtain gas pressure: 30 psi; collision gas: high; ion source gas 1 pressure: 50 psi; and ion source gas 2 pressure: 50 psi. Chromatographic separation was performed using a gradient elution program of mobile phases A (0.05% formic acid in water) and B (0.05% formic acid in methanol) at a flow rate of 0.300 mL/min on a Raptor Biphenyl column (2.1 mm × 100 mm, 1.7 μm particles) maintained at 60 °C. The gradient was set as follows: 25 to 50% B over 3 min, 50 to 55% B for 8 min, 55 to 100% B for 13 min, and 13 to 13.1 min decreasing to starting conditions (25% eluent B), which were maintained until the end of the run at 15 min.

### 2.5. Sample Preparation

#### 2.5.1. Extraction Procedure (Step 1)

The sample weight can influence the amount of solvent or reagent used during the analysis. To obtain a suitable sample weight and extraction solvent volume, recovery experiments were conducted at low and high concentrations under the following conditions: (1) 250 mg and 2 mL; (2) 500 mg and 4 mL; and (3) 1000 mg and 8 mL. The results are presented in Figure S1 (Supplementary Material). This analysis was based on a mycotoxin extraction method [11]. The sample weight was set to 250 mg, considering the recovery rate and the amount of solvent and reagents used.

The sample was weighed to 250 mg and placed in a 5 mL polypropylene tube. The sample was then dispersed with 2 mL of an acetonitrile/water solution (80:20, *v*/*v*) containing 0.5% formic acid and agitated for 1 min using the Vortex-Genie 2T (Scientific Industries, Bohemia, NY, USA). The sample was then shaken for 20 min on a rotary shaker (MyLab SLRM-3, SeouLin Bioscience, Seongnam, Republic of Korea) and centrifuged at 9190× *g* and 4 °C for 10 min using a high-speed centrifuge (Himac CR22N; Hitachi Ltd., Tokyo, Japan). The supernatant was transferred in 1 mL aliquots to 1.5 mL polypropylene tubes packed with various d-SPE sorbents.

#### 2.5.2. Sample Cleanup with Dispersive SPE Sorbent (Step 2)

The extract was purified via the d-SPE method using various sorbents such as silica gel, Florisil, C_18_ EC, PSA, Al_2_O_3_, and Z-sep. Briefly, 1 mL of the sample extract was added to a 1.5 mL tube containing the d-SPE sorbent, vigorously agitated for 1 min, and then centrifuged at 15,294× *g* and 4 °C for 10 min using a bench centrifuge (5810/5810 R; Eppendorf, Leipzig, Germany). The supernatant (800 μL) was evaporated to dryness at 40 °C under a gentle stream of nitrogen. The dried extract was then reconstituted with 400 μL of a water/methanol solution (50:50, *v*/*v*) and filtered through a 0.2 μm PTFE filter cartridge (Restek Corp., Bellefonte, PA, USA). The final solution was prepared for UPLC-MS/MS analysis. C_18_ EC was selected as the d-SPE sorbent for the study, considering its recovery and matrix effects (MEs). Additionally, the d-SPE system was cleaned with C_18_ EC at five different concentrations (10, 25, 50, 100, and 150 mg) to obtain the best cleanup performance.

### 2.6. Method Validation

The optimized method was validated according to the guidelines established by the European Commission Decision 2002/657/EC for identification, linearity, the LOD, the LOQ, accuracy, repeatability, and in-laboratory reproducibility. The identification and confirmation of each compound were assessed using the retention times and relative ion ratios of the selected MRM transitions. Linearity was measured using matrix-matched calibration curves at six concentration levels. The LODs and LOQs were calculated to be 3.3 and 10 times the standard deviation of the intercept/slope of the calibration curve, respectively. Accuracy was assessed by analyzing blank samples spiked with the standard solution at three concentration levels, hereinafter referred to as “low”, “medium”, and “high” (for AFs, OTs, FBs, and EAs: 5, 10, and 20 μg/kg; for trichothecenes except for NIV: 25, 50, and 100 μg/kg; for NIV: 50, 100, and 200 μg/kg), then identifying the recovery (%). Repeatability and reproducibility were determined using blank samples at the same concentration levels as above; measurements were conducted five times within one day and over three consecutive days, and the data were expressed in terms of the relative standard deviation (RSD). To evaluate the MEs, we compared the mean area responses of the standard solutions and the blank samples spiked with analytes after extraction.

### 2.7. Measurement Uncertainty

Measurement uncertainty was estimated based on the EURACHEM guide [18]. The sources of uncertainty were identified as the sample weight (weight), extraction solvent volume (vol), purity of the reference material (RM), interpolation of the linear calibration curve (cal), and repeatability (rep). The calibration curve used was a matrix-matched calibration curve, and repeatability was assessed based on recovery by adding high concentrations of the analytes to blank samples. The expanded uncertainty (*U*_exp_) was derived using Equation (1), which involved multiplying the aggregate standard uncertainty with a coverage factor of *k* = 2, achieving an approximate confidence level of 95%.

(1) $$U_{exp}=\sqrt{u_{weight}^{2}+u_{vol}^{2}+u_{RM}^{2}+u_{Cal}^{2}+u_{rep}^{2}}\times k\;$$

### 2.8. Stability

The stability of the analytes in the blank samples under different storage conditions was tested by analyzing the samples in triplicate at both low and high concentrations. The samples were exposed to temperatures of 4 and −20 °C for 1, 3, and 7 days.

### 2.9. Application to Real Samples

The developed method was applied to the simultaneous detection of multiple mycotoxins in cereal samples from the local market. Sample determination was performed in quintuplicate, and the analysis was based on matrix-matched calibration curves.

### 2.10. Statistical Analysis

Quantitative calculations for the target analytes and statistical analyses were performed using MS Office Excel 2016 (Microsoft Corporation, Redmond, WA, USA).

## 3. Results and Discussion

### 3.1. Optimization of UPLC–MS/MS Analysis Conditions

The optimal MRM parameters were obtained through tuning experiments, with individual mycotoxins being directly infused at concentrations of 10–100 μg/L. A comparison between the positive and negative ion modes indicated that operating in the positive mode resulted in the formation of more high-intensity product ions for 33 mycotoxins, except for NIV and ZEA. In the optimization of mycotoxins using full-scan MS, the most intense ions were identified as [M+H]^+^ for all mycotoxins except T-2 and HT-2 ([M+NH_4_]^+^), NIV ([M+HCOO]^−^), and ZEA ([M−H]^−^). Ammonia was not added to the mobile phases, but it may have been generated in the source through redox processes during the electrospray process or present in the solvents or from the previous experiment [19,20]. Similar findings have been reported in other studies [21,22]. The MS/MS parameters are listed in Table 1.

**Figure 1:**
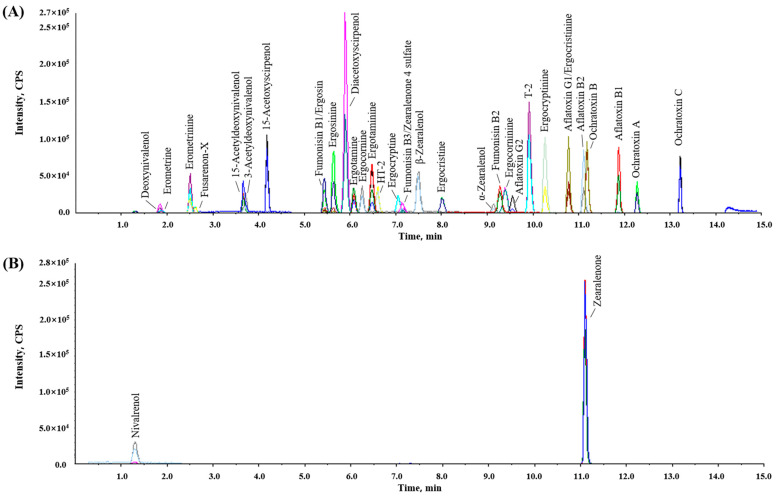
Total multiple-reaction monitoring chromatograms of mycotoxins and their derivatives. (**A**) Positive mode and (**B**) negative mode.

LC separation was performed as described by [11] with slight modifications. The stationary phase of the biphenyl column separated and recorded a good peak shape for the target mycotoxins with an LC gradient program of 15 min in LC-MS/MS. Additionally, to improve the tailing of the chromatographic peak, 0.05% formic acid was added to both the aqueous and organic phases; methanol was chosen as the organic phase because of its superior separation efficiency compared to acetonitrile. Figure 1 shows the LC-MS/MS MRM chromatograms of a working solution containing the 35 target mycotoxins. The optimized MRM transitions presented higher sensitivity and selectivity for the mycotoxins at 0.25 to 100 μg/kg in the blank sample matrix.

### 3.2. Optimization of the Cleanup Procedure

**Figure 2:**
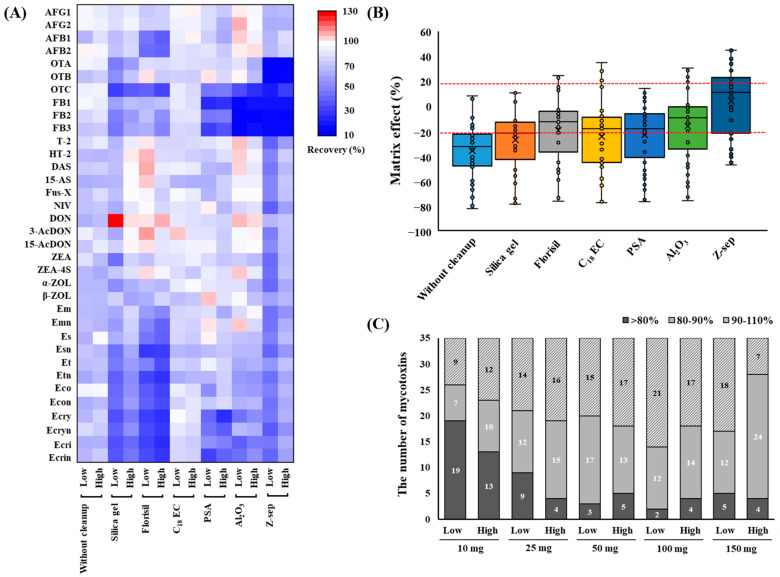
Heatmap of the recoveries (**A**) and matrix effects (**B**) for the mycotoxins and their derivatives in blank samples using different purifying sorbents. The recovery efficiencies of different additive amounts of C_18_EC (10, 25, 50, 100, and 150 mg) for the mycotoxins and their derivatives (**C**). AFG1, aflatoxin G1; AFG2, aflatoxin G2; AFB1, aflatoxin B1; AFB2, aflatoxin B2; OTA, ochratoxin A; OTB, ochratoxin B; OTC, ochratoxin C; FB1, fumonisin B1; FB2, fumonisin B2; FB3, fumonisin B3; T-2, T-2 toxin; HT-2, HT-2 toxin; DAS, diacetoxyscirpenol; 15-AS, 15-acetoxyscirpenol; Fus-X, fusarenon X; NIV, nivalenol; DON, deoxynivalenol; 3-AcDON, 3-acetyl deoxynivalenol; 15-AcDON, 15-acetyl deoxynivalenol; ZEA, zearalenone; ZEA-4S, zearalenone 4-sulfate; α-ZOL, α-Zearalenol; β-ZOL, β-Zearalenol; Em, ergometrine; Emn, ergometrinine; Es, ergosine; Esn, ergosinine; Et, ergotamine; Etn, ergotaminine; Eco, ergocornine; Econ, ergocorninine; Ecry, ergocryptine; Ecryn, ergocryptinine; Ecri, ergocristine; Ecrin, ergocristinine.

The results of the purification of the blank sample extract containing mycotoxins are shown in Figure 2. Commercially available d-SPE sorbents such as silica gel, Florisil, C_18_EC, PSA, Al_2_O_3_, and Z-sep clearly differed in their abilities to purify various substance classes. Figure 2A shows a heatmap of the recovery rates of the mycotoxins. When C_18_EC was used for cleanup, most mycotoxins were distributed close to white in the heatmap, indicating recoveries between 70% and 101% (see Table S2 for details). C_18_EC is known to provide high recoveries for both apolar and polar analytes by effectively removing nonpolar interfering substances, lipids, and sterols from the sample matrices [23,24]. Florisil is used for the separation of hydrophilic substances from non-aqueous nonpolar mixtures for the analysis of samples with high contents of sugars, acids, pigments, and organic ingredients [25]. Notably, the use of Florisil resulted in a lower recovery rate of AFs and EAs than those obtained with other sorbents, indicating that it had a strong absorption affinity for the polar groups in the structures of the target mycotoxins. This suggests that AFs, which represent a polar class of mycotoxins, were irreversibly adsorbed onto Florisil, leading to them not being recovered. Similar results were observed for several other mycotoxins (AFs, Et, Eco, Ecri, and Es), with low recoveries obtained owing to them being strongly absorbed by Florisil [26]. Furthermore, 50 mg of Florisil was used during the d-SPE of grains, indicating that the recovery of AFB1 was less than 40% [27]. The results for silica gel showed that the recoveries of some analytes such as OTs, FBs, and EAs were <72% at low concentrations. However, when the three materials with acidic interference removal functions (PSA, Al_2_O_3_, and Z-sep) were used for cleanup, the recovery of the FBs was lower than 50%. This inadequate recovery can be attributed to the acidic nature of the carboxylic groups in the FBs, which led to their adsorption onto the cleanup sorbents. In a study performed by Mateus [28] on pistachio nuts, FB1 and FB2 were similarly not detected by PSA and Z-sep, whereas C_18_ provided analytical signals for all 10 mycotoxins tested.

MEs were assessed for the different cleanup procedures. As shown in Figure 2B, the results obtained from the six tested sorbents after cleanup showed a reduction in the average ME% compared to those obtained without cleanup from the blank sample extract (see Table S2 for details). In the case of cleanup using Z-Sep+, the range of MEs was reduced to −47% to 45% compared to the wheat extract without cleanup. These results correspond with the color change observed in the precipitates after d-SPE cleanup; compared with the other sorbents, Z-sep showed a stronger yellowish discoloration. However, this led to a significant reduction in the recovery of OTs and FBs; therefore, Z-sep was not considered. Based on these results, C_18_EC was selected as the cleanup sorbent for this study. In addition, the amounts of C_18_EC (10, 25, 50, 100, and 150 mg) used for the target mycotoxins were optimized. The results of the comparison are shown in Figure 2C. As the amount of C_18_EC increased, the number of mycotoxins that could be recovered to within 80–110% also increased. However, when 150 mg of C_18_EC was used, the number of mycotoxins showing < 80% recovery at low concentrations increased compared to when 100 mg was used. Therefore, 100 mg of C_18_EC was selected as the final cleanup condition because of its high recovery efficiency and effective cleanup performance.

### 3.3. Analytical Method Validation

#### 3.3.1. Linearity and Detectability

The linearity of the matrix-matched calibration curve was in the range of 0.25–10 μg/kg for AFs, OTs, FBs, and EAs, 1.25–50 μg/kg for trichothecenes, and 2.5–100 μg/kg for NIV. The calibration curves showed good linearity for all the target mycotoxins, with correlation coefficient (R^2^) values higher than 0.997. The linear equations for all analytes in the blank samples are provided in the Supplementary Material (Table S3).

Detectability was evaluated using LOD and LOQ values. The LODs of the analytes were 0.01–7.50 µg/kg. The LOQs for mycotoxins in the wheat flour matrix ranged over 0.02–22.73 µg/kg, based on the acceptable recovery (70–120%) for all mycotoxins; the values were lower than the MLs proposed by the European Commission Regulation (EU) 2023/915 for AFs (2–4 µg/kg), DON (750–1750 µg/kg), ZEN (50–100 µg/kg), OTA (3–5 µg/kg), FB1 and B2 (800 µg/kg), and EAs (50–250 µg/kg) for all cereals and related products [29]. The detectability of the analytical method was found to surpass that of previous LC-MS/MS methods used for analyzing 23 mycotoxins in wheat, with an LOQ range of 0.12 to 5.84 μg/kg [30]. Furthermore, these LODs and LOQs were lower than those obtained in previous studies via QuEChERS-UHPLC-MS/MS for the detection of 42 mycotoxins from oats [31] and HPLC-MS/MS for the detection of EAs from cool-season-adapted cereal grains [32]. The detailed results are presented in Table 2.

#### 3.3.2. Accuracy and Precision

The accuracy of the method was assessed using a mixture of target mycotoxins in the blank samples at three concentration levels (low, middle, and high); the recoveries were in the ranges of 82.6–107.2%, 81.3–104.0%, and 80.4–101.1%, respectively. Precision was determined by analyzing five replicates of each of the three concentrations of the fortified samples within a single day (intra-day) and over three consecutive days (inter-day) (Table 2). The RSDs were between 0.8% and 9.2% for intra-day results and between 2.2% and 12.2% for inter-day results, which are below the acceptable value of RSD < 15%, indicating the acceptable repeatability and reproducibility of the analysis method. Varga et al. [33] reported that the precision of the method for analyzing 12 mycotoxins in flour, intra-day, ranged from 1.3% to 8.8%, and the inter-day precision ranged from 7.7% to 17.1%. This demonstrated a relatively lower precision compared to the present study.

These results show that this analytical method is suitable for the simultaneous analysis of multiple mycotoxins in cereal samples.

#### 3.3.3. Matrix Effect

Interference from endogenous substances causes MEs, which can enhance or diminish the analyte signal and affect the accuracy and precision of the analytical results. Table 2 shows that 28 mycotoxins exhibited negligible or medium MEs, varying between −50% and 50%. In contrast, AFB1, AFB2, NIV, 3-AcDON, 15-AcDON, ZEA, and ZEA-4S toxins displayed stronger MEs (suppression of over 50%), as they had a greater vulnerability to matrix interference compounds. Similarly, AFB1, AFB2, NIV, 3-AcDON, and 15-AcDON have been reported to suppress ions in durum wheat samples [34]. Therefore, in this study, matrix-matched calibration was used to compensate for the external standard to solve the problem caused by the ME of mismeasurement.

### 3.4. Estimation of Measurement of Uncertainty

Uncertainty is an important metric for setting the permissible error range in test standards and judging the reliability of the results. The measurement uncertainty was estimated as the expanded uncertainty (*U*_exp_) owing to sample recovery upon spiking the sample with a specific concentration of the mycotoxin mixture (Table 2). The *U*_exp_ value for mycotoxins in wheat varied between 1.6 and 11.4%. The contributions of each uncertainty factor to the expanded uncertainty (*U*_exp_) value are shown in Figure S2. According to the European Commission guidelines, acceptable expanded uncertainty (*U*_exp_) values are <44% when the sample concentration is less than 100 μg/kg [35]. These results indicated that the uncertainty of the established method was adequate for this application.

### 3.5. Stability of Mycotoxins and Their Derivatives

Stability studies revealed that all 35 mycotoxins and their derivatives were stable under 4 °C and −20 °C storage conditions for at least 3 days, reaching recoveries between 80.0% and 110.0% for analytes spiked at low concentrations, and between 80.7% and 104.8% for analytes spiked at high concentrations (Table S4). Although most of the mycotoxins could be stably stored for 7 days at 4 °C and −20 °C, OTC, Esn, Econ, α-ZOL, and NIV exhibited below-acceptable recovery (less than 70–120%) at 4 °C after 7 days. Therefore, it is recommended that all compounds reconstituted in 50% methanol after extraction and purification be analyzed within 3 days when kept in an autosampler at 4 °C.

### 3.6. Application to the Analysis of Real Samples

A validated analytical method was used to test 23 cereal samples including rye flour, whole-wheat flour, and wheat flour for the presence of mycotoxins. All samples were stored at −20 °C after purchase and analyzed in February 2024. Detailed information on the samples is provided in Table S1. Of the 23 cereal samples tested, 17 samples, that is, 74% of the total samples, showed positive results for contamination with one or more mycotoxins. The following mycotoxins were detected as contaminants in the cereal samples: OTA (<LOQ–0.19 μg/kg), T-2 (<LOQ–0.71 μg/kg), HT-2 (<LOQ–11.69 μg/kg), DON (<LOQ–204.85 μg/kg), 3-AcDON (<LOQ–11.55 μg/kg), EAs (0.42–609.63 μg/kg), and ZEA (<LOQ–11.01 μg/kg). However, except for 2 out of the 17 samples (11.8%), the detected levels of regulated mycotoxins did not surpass established MLs. EAs were detected in most samples (56%), and the two exceeding samples exhibited EA contamination at concentrations of 609.63 μg/kg (S1) and 294.93 μg/kg (S6), respectively, which exceed the 250 μg/kg ML set for rye milling products [29]. In particular, regarding the S1 sample produced in 2021, it is difficult to determine whether the high contamination level is due to initial *Claviceps purpurea* contamination or changes that occurred during the storage process. Cherewyk [36] reported that EAs increase or decrease under different storage times (0, 1, 2, and 4 months) and temperatures (room temperature, 4 °C, and −20 °C). Although DON was the most frequently detected in our samples, the concentrations were far below the MLs set by the EC for unprocessed and processed cereal (600–1000 µg/kg) [29].

As shown in Table 3, the co-occurrence of mycotoxins was observed in 11 out of the total 23 samples analyzed (48%, 11/23). The highest co-occurrence was observed in S5, which contained T-2, HT-2, DON, EAs, and ZEA. Similarly, a recent study reported the co-contamination of wheat samples with EAs, FBs (FB1 and FB2), T-2, ZEA, DON, and their derivatives (3-AcDON and 15-AcDON) [12]. The most common paired combinations were DON + EAs (73%, 8/11), T-2 + HT-2 (45%, 5/11), OTA + DON (36%, 4/11), T-2 + HT-2 + DON (36%, 4/11), and T-2 + HT-2 + EAs (36%, 4/11). Following recent studies on the combined toxicological effects of mycotoxins in model systems, the presence of co-occurring mycotoxins may raise concerns [37]. Therefore, continued monitoring studies are warranted to characterize the co-contamination patterns of multiple mycotoxins in marketed foods. This will further our understanding of mycotoxin interactions and the health risks associated with chronic dietary exposure.

## 4. Conclusions

A novel method based on d-SPE cleanup and UPLC-MS/MS was developed for the simultaneous determination of 35 mycotoxins and their derivatives in cereals. The C_18_EC sorbent demonstrated superior performance in terms of cleanup efficiency and yielded the highest recovery for the selected mycotoxins. The optimized method demonstrated good linearity, sensitivity, accuracy, and repeatability, as evidenced by calibration curves and recovery assessments. The proposed method offers a simple, reliable, and rapid approach, requiring a minimal sample (250 mg) and organic solvent (2 mL) and approximately 15 min to analyze 35 mycotoxins in a cereal sample. Furthermore, the methodology could simultaneously detect OTA, T-2, HT-2, DON, 3-AcDON, EAs, and ZEA in cereal samples. In addition, 23 cereal samples were analyzed; contamination was detected in 74% of the samples, including EAs, which were previously not expected to be detected as they are mainly observed in Europe. This analytical method was developed, validated, and applied for cereal powders. For processed cereal products, matrix effects from various additives (sugar, protein, fat, food colorings, etc.) may differ compared to the powder form. Therefore, further research is needed on combinations of sorbents that can remove proteins, lipids, or other interfering compounds, along with C18 sorbents.

## Figures and Tables

**Table 1:** Mass ion transitions, parameters, and respective retention times of the target analytes.

Analyte ^a^	Formula	RT (min)	Adduct	Precursor ion (*m*/*z*)	Production (*m*/*z*) ^b^	DP (V) ^c^	CE (V) ^d^	CXP (V) ^e^
AFG1	C_17_H_12_O_7_	10.8	[M+H]^+^	328.9	242.9/199.9	106	39/55	8/6
AFG2	C_17_H_14_O_7_	9.3	[M+H]^+^	331.0	312.9/244.8	121	35/43	66
AFB1	C_17_H_12_O_6_	11.8	[M+H]^+^	313.0	285.0/213.0	116	35/61	8/6
AFB2	C_17_H_14_O_6_	11.1	[M+H]^+^	315.0	258.9/287.3	101	41/39	4/24
OTA	C_20_H_18_ClNO_6_	12.3	[M+H]^+^	404.0	238.8/220.9	81	35/51	6/6
OTB	C_20_H_19_NO_6_	11.2	[M+H]^+^	370.0	204.9/187.2	56	31/51	6/10
OTC	C_22_H_22_ClNO_6_	13.2	[M+H]^+^	432.0	239.2/358.2	111	41/25	8/12
FB1	C_34_H_59_NO_15_	5.2	[M+H]^+^	722.3	334.2/352.1	126	57/51	10/10
FB2	C_34_H_59_NO_14_	8.8	[M+H]^+^	706.2	336.4/688.2	141	51/41	12/20
FB3	C_34_H_59_NO_14_	6.9	[M+H]^+^	706.3	336.1/81.2	111	51/111	10/8
T-2	C_24_H_34_O_9_	9.9	[M+NH_4_]^+^	484.2	305.2/215.1	71	21/25	12/6
HT-2	C_22_H_32_O_8_	6.6	[M+NH_4_]^+^	442.2	263.1/215.1	6	19/19	8/6
DAS	C_19_H_26_O_7_	5.9	[M+H]^+^	384.1	307.2/247.2	1	17/19	10/10
15-AS	C_17_H_24_O_6_	4.2	[M+NH_4_]^+^	342.1	265.4/107.2	16	13/25	10/14
Fus-X	C_17_H_22_O_8_	2.6	[M+H]^+^	355.1	247.2/175.3	66	15/27	10/12
NIV	C_15_H_20_O_7_	1.3	[M+HCOO]^−^	357.0	281.0/310.9	−5	−18/−14	−9/−11
DON	C_15_H_20_O_6_	1.8	[M+H]^+^	297.1	249.2/203.2	76	17/21	10/6
3-AcDON	C_17_H_22_O_7_	3.7	[M+H]^+^	339.1	231.3/203.1	71	13/17	10/8
15-AcDON	C_17_H_22_O_7_	3.6	[M+H]^+^	339.1	321.1/261.1	16	13/25	14/8
ZEA	C_18_H_22_O_5_	11.0	[M-H]^−^	317.0	174.9/131.0	−105	−34/−38	−9/−7
ZEA-4S	C_18_H_25_NO_8_S	7.2	[M+H]^+^	416.1	319.2/187.2	66	17/41	10/6
α-ZOL	C_18_H_24_O_5_	9.3	[M+H]^+^	321.2	303.0/285.0	51	11/17	12/8
β-ZOL	C_18_H_24_O_5_	7.5	[M+H]^+^	321.1	303.1/285.4	56	11/17	8/8
Em	C_19_H_23_N_3_O_2_	1.9	[M+H]^+^	326.1	206.9/222.9	76	59/33	6/6
Emn	C_19_H_23_N_3_O_2_	2.5	[M+H]^+^	325.8	208.0/207.1	131	39/59	6/6
Es	C_30_H_37_N_5_O_5_	5.4	[M+H]^+^	548.0	223.0/208.1	81	45/59	6/6
Esn	C_30_H_37_N_5_O_5_	5.6	[M+H]^+^	547.8	222.9/152.0	131	45/165	6/4
Et	C_33_H_35_N_5_O_5_	6.1	[M+H]^+^	582.0	223.0/207.9	91	47/65	6/6
Etn	C_33_H_35_N_5_O_5_	6.5	[M+H]^+^	582.1	223.0/208.1	86	47/69	6/6
Eco	C_31_H_39_N_5_O_5_	6.3	[M+H]^+^	562.0	268.0/223.0	96	37/51	8/6
Econ	C_31_H_39_N_5_O_5_	9.4	[M+H]^+^	562.4	223.0/227.0	1	51/39	6/8
Ecry	C_32_H_41_N_5_O_5_	7.1	[M+H]^+^	576.0	267.9/208.0	106	37/67	8/6
Ecryn	C_32_H_41_N_5_O_5_	10.3	[M+H]^+^	576.1	222.9/305.3	6	51/39	6/10
Ecri	C_35_H_39_N_5_O_5_	7.6	[M+H]^+^	610.1	208.0/223.0	96	65/55	6/6
Ecrin	C_35_H_39_N_5_O_5_	10.5	[M+H]^+^	609.9	223.0/305.2	1	55/39	6/10

^a^ AFG1, aflatoxin G1; AFG2, aflatoxin G2; AFB1, aflatoxin B1; AFB2, aflatoxin B2; OTA, ochratoxin A; OTB, ochratoxin B; OTC, ochratoxin C; FB1, fumonisin B1; FB2, fumonisin B2; FB3, fumonisin B3; T-2, T-2 toxin; HT-2, HT-2 toxin; DAS, diacetoxyscirpenol; 15-AS, 15-acetoxyscirpenol; Fus-X, fusarenon X; NIV, nivalenol; DON, deoxynivalenol; 3-AcDON, 3-acetyl deoxynivalenol; 15-AcDON, 15-acetyl deoxynivalenol; ZEA, zearalenone; ZEA-4S, zearalenone 4-sulfate; α-ZOL, α-Zearalenol; β-ZOL, β-Zearalenol; Em, ergometrine; Emn, ergometrinine; Es, ergosine; Esn, ergosinine; Et, ergotamine; Etn, ergotaminine; Eco, ergocornine; Econ, ergocorninine; Ecry, ergocryptine; Ecryn, ergocryptinine; Ecri, ergocristine; Ecrin, ergocristinine. ^b–e^ Quantifier ion/qualifier ion. DP: declustering potential; CE: collision energy; CXP: collision cell exit potential.

**Table 2:** Validation characteristics of the optimized strategy for the simultaneous analysis of multiple mycotoxins and their derivatives in cereals.

Analyte ^a^	Calibration Range (μg/kg)	Matrix-Matched Calibration (R^2^)	Matrix Effect (%)	LOD ^b^ (μg/kg)	LOQ ^c^ (μg/kg)	U ^d^ (%)	Intra-Day (*n* = 5)						Inter-Day (*n* = 15)		
							Low ^e^		Medium ^f^		High ^g^		Low	Medium	High
							RE (%) ^h^	RSD_r_	RE (%)	RSD_r_	RE (%)	RSD_r_	RSD_R_	RSD_R_	RSD_R_
AFG1	0.25–10	0.9995	−48.23	0.14	0.43	10.90	93.33	3.83	91.86	4.60	91.24	2.48	8.65	4.65	5.04
AFG2	0.25–10	0.9996	−24.36	0.06	0.17	10.64	92.77	5.84	87.58	4.29	85.92	1.61	6.52	4.34	5.36
AFB1	0.25–10	0.9997	−64.61	0.04	0.13	11.33	101.43	2.30	98.97	1.21	95.24	2.21	5.84	7.54	6.71
AFB2	0.25–10	0.9999	−64.06	0.02	0.06	11.43	103.16	6.21	104.02	5.91	101.1	3.79	10.42	9.27	8.94
OTA	0.25–10	0.9997	−48.63	0.03	0.08	10.47	97.15	5.94	91.80	0.88	88.93	4.56	7.77	5.34	6.52
OTB	0.25–10	0.9998	−8.14	0.01	0.04	11.11	97.10	5.00	94.17	4.22	91.06	2.88	4.62	3.57	5.11
OTC	0.25–10	0.9998	17.31	0.04	0.12	9.49	85.01	1.00	84.83	2.21	86.00	1.91	2.59	3.07	3.24
FB1	0.25–10	0.9999	31.57	0.10	0.30	10.95	92.21	7.48	89.56	2.08	88.32	1.76	6.46	13.77	4.63
FB2	0.25–10	0.9998	27.32	0.03	0.09	10.05	87.88	3.69	85.55	3.39	80.39	3.53	4.63	5.34	6.18
FB3	0.25–10	0.9995	13.53	0.37	1.12	10.55	98.04	5.44	89.67	3.09	88.74	3.14	6.68	8.84	4.06
T-2	1.25–50	0.9996	−2.62	0.15	0.45	5.55	94.21	4.30	91.61	2.95	89.43	1.92	5.42	3.73	4.18
HT-2	1.25–50	0.9994	−21.50	0.41	1.23	5.23	107.16	1.83	98.61	2.81	95.88	4.00	4.55	6.31	4.82
DAS	1.25–50	0.9999	15.39	0.12	0.37	5.55	98.12	1.85	96.44	1.92	94.06	1.70	4.03	2.51	3.10
15-AS	1.25–50	0.9998	−18.12	0.14	0.43	4.74	100.73	1.82	95.56	2.87	95.13	1.98	3.52	4.51	3.44
Fus-X	1.25–50	0.9996	−35.65	0.76	2.31	5.48	95.72	8.06	95.13	5.08	95.51	3.26	9.10	5.77	5.85
NIV	2.5–100	0.9992	−76.67	7.50	22.73	1.62	98.89	7.26	91.72	4.38	84.98	1.79	9.51	6.97	6.20
DON	1.25–50	0.9989	−29.86	3.38	10.23	5.27	85.78	4.77	88.42	10.41	95.75	10.12	10.16	11.40	9.56
3-AcDON	1.25–50	0.9993	−76.84	2.85	8.65	5.36	99.22	4.53	100.19	4.33	96.90	5.24	7.26	10.99	9.17
15-AcDON	1.25–50	0.9990	−75.69	2.54	7.70	5.01	97.52	8.26	89.89	4.23	96.53	5.68	9.42	4.91	7.71
ZEA	1.25–50	0.9997	−59.09	0.39	1.19	5.37	93.44	4.10	88.21	1.09	84.35	5.80	8.43	8.30	10.37
ZEA-4S	1.25–50	0.9995	−63.46	0.20	0.59	4.94	98.20	8.60	95.41	3.10	90.34	5.39	8.52	6.76	11.70
α-ZOL	1.25–50	0.9967	−9.72	0.84	2.53	4.94	82.60	5.55	86.26	2.27	92.53	2.45	12.13	7.33	4.46
β-ZOL	1.25–50	0.9976	−0.28	2.11	6.41	4.94	88.56	6.10	91.54	4.20	85.28	1.05	12.20	9.34	8.46
Em	0.25–10	0.9988	−20.22	0.39	1.17	10.72	88.50	6.82	98.62	8.93	87.33	7.40	12.02	11.03	9.03
Emn	0.25–10	0.9999	−16.70	0.10	0.29	10.03	86.94	7.77	88.28	3.75	88.43	2.54	9.38	5.65	3.96
Es	0.25–10	0.9997	−27.19	0.01	0.03	10.73	98.10	5.42	100.17	4.64	97.99	4.51	3.82	5.06	4.45
Esn	0.25–10	0.9996	−11.26	0.04	0.12	9.88	93.86	7.36	86.83	4.82	80.55	4.31	10.86	10.78	8.29
Et	0.25–10	0.9997	−12.52	0.12	0.37	9.88	97.93	6.09	94.95	7.67	95.41	7.94	6.70	8.29	6.51
Etn	0.25–10	0.9998	−21.30	0.02	0.06	9.88	85.43	5.34	84.65	2.50	84.93	3.54	9.33	4.89	5.86
Eco	0.25–10	0.9998	−14.68	0.02	0.05	9.05	97.83	6.34	92.81	7.45	84.61	5.93	14.20	9.32	10.79
Econ	0.25–10	0.9997	−10.92	0.07	0.22	9.52	86.42	2.07	84.85	1.99	81.07	3.93	4.26	3.16	4.37
Ecry	0.25–10	0.9994	−30.01	0.10	0.29	9.92	92.42	8.01	87.63	5.27	83.09	6.76	10.08	5.59	11.40
Ecryn	0.25–10	0.9987	−45.35	0.46	1.40	9.50	85.10	6.13	81.65	3.13	84.94	2.78	7.97	4.89	8.25
Ecri	0.25–10	0.9998	−9.55	0.01	0.02	10.07	82.86	5.54	81.34	2.21	83.73	6.38	9.28	12.65	12.66
Ecrin	0.25–10	0.9999	−43.34	0.05	0.15	9.72	84.50	6.30	81.95	2.84	81.71	2.69	8.85	9.41	8.80

^a^ AFG1, aflatoxin G1; AFG2, aflatoxin G2; AFB1, aflatoxin B1; AFB2, aflatoxin B2; OTA, ochratoxin A; OTB, ochratoxin B; OTC, ochratoxin C; FB1, fumonisin B1; FB2, fumonisin B2; FB3, fumonisin B3; T-2, T-2 toxin; HT-2, HT-2 toxin; DAS, diacetoxyscirpenol; 15-AS, 15-acetoxyscirpenol; Fus-X, fusarenon X; NIV, nivalenol; DON, deoxynivalenol; 3-AcDON, 3-acetyl deoxynivalenol; 15-AcDON, 15-acetyl deoxynivalenol; ZEA, zearalenone; ZEA-4S, zearalenone 4-sulfate; α-ZOL, α-Zearalenol; β-ZOL, β-Zearalenol; Em, ergometrine; Emn, ergometrinine; Es, ergosine; Esn, ergosinine; Et, ergotamine; Etn, ergotaminine; Eco, ergocornine; Econ, ergocorninine; Ecry, ergocryptine; Ecryn, ergocryptinine; Ecri, ergocristine; Ecrin, ergocristinine. ^b–d^ LOD, limit of detection; LOQ, limit of quantitation; U, expanded uncertainty. ^e–g^ Low level, 5 μg/kg for AFs, OTs, FBs and EAs, 25 μg/kg for T-2, HT-2, DAS, 15-AS, Fus-X, DON, 3-AcDON, 15-AcDON, ZEA, ZEA-4S, α-ZOL, and β-ZOL, 50 μg/kg for NIV; medium level, 2 times that of low level; high level, 4 times that of low level. ^h^ Recovery (%).

**Table 3:** Occurrence and concentration of mycotoxins (μg/kg) in cereal samples.

Samples	OTA ^a^	T-2	HT-2	DON	3-AcDON	EAs	ZEA	Number of Detected Mycotoxins
S1	– ^a^	–	–	–	–	609.63	–	1
S2	–	–	–	204.85	<LOQ ^b^	3.70	11.01	4
S4	–	<LOQ	7.37	–	–	16.85	–	3
S5	–	<LOQ	9.79	82.05	–	202.91	<LOQ	5
S6	–	0.71	11.69	119.76	–	294.93	–	4
S7	–	<LOQ	9.10	47.68	–	5.27	–	4
S8	–	–	–	–	<LOQ	72.55	–	2
S9	0.19	–	–	79.34	11.55	11.55	–	4
S13	–	–	–	22.93	–	–	–	1
S15	<LOQ	–	–	13.73	–	11.97	–	3
S17	0.13	0.64	<LOQ	<LOQ	–	–	–	4
S18	–	–	–	18.78	–	–	–	1
S19	–	–	–	–	–	2.97	–	1
S20	–	–	–	–	–	1.25	–	1
S21	–	–	–	<LOQ	–	2.29	–	2
S22	–	–	–	–	–	0.42	–	1
S23	<LOQ	–	–	<LOQ	–	10.11	–	3

^a^ OTA: ochratoxin A; T-2: T-2 toxin; HT-2: HT-2 toxin; DON: deoxynivalenol; 3-AcDON: 3-acetyl deoxynivalenol; EAs: ergot alkaloids; ZEA: zearalenone. ^b^ –, not detected; <LOQ, less than the limit of quantification.

## Data Availability

The original contributions presented in this study are included in the article/Supplementary Materials. Further inquiries can be directed to the corresponding author.

## Supplementary Materials

The following supporting information can be downloaded at https://www.mdpi.com/article/10.3390/foods13223617/s1. Table S1: Sample information. Figure S1: The recoveries of different sample weights and extraction solvent volumes for LC-MS/MS at low (5 μg/kg for AFs, OTs, FBs, and EAs, 25 μg/kg for T-2, HT-2, DAS, 15-AS, Fus-X, DON, 3-AcDON, 15-AcDON, ZEA, ZEA-4S, α-ZOL, and β-ZOL, 50 μg/kg for NIV) and high fortification levels (4 times that of the low level). Figure S2: Contribution of each parameter of uncertainty in wheat (u_weight_: sample weight; u_vol_: extraction solvent volume; u_RM_: reference material; u_cal_: calibration curve; u_rep_: repeatability). Table S2: Recoveries and matrix effects of mycotoxins in the alternative cleanup sorbents (at low and high fortification levels). Table S3: Linear range and linear equation of multiple mycotoxins and derivatives in cereals. Table S4: Stability result of mycotoxins in wheat under different storage conditions (n = 3).
