# Supplementary material for: Optimization, Validation, and Application of Cleanup-Coupled Liquid Chromatography–Tandem Mass Spectrometry for the Simultaneous Analyses of 35 Mycotoxins and Their Derivatives in Cereals
Source: Foods. 2024 Nov 13;13(22):3617. doi: 10.3390/foods13223617 (PMC11594120; doi:10.3390/foods13223617)
Supplement: Supplementary file 1 [file foods-13-03617-s001.zip › foods-3249904-supplementary.pdf]

Supplementary Data

Optimization, Validation, and Application of Cleanup-Coupled Liquid Chromatography–Tandem Mass Spectrometry for the Simultaneous Analyses of 35 Mycotoxins and Their Derivatives in Cereals

Dan-Bi Kim [1,2], Miso Nam [1], Yong-Suk Kim [2] and Min-Sun Kim [1,*]

[1] Food Analysis Research Center, Korea Food Research Institute, Wanju 55365, Republic of Korea; dbkim1022@kfri.re.kr (D.-B.K.); msnam@kfri.re.kr (M.N.)
[2] Department of Food Science and Technology, Jeonbuk National University, Jeonju 54896, Republic of Korea;
* Correspondence: mskim@kfri.re.kr

Table S1. Sample information.

Sample name	Raw material	Processed form	Origin	Date of production	Use-By date[a]
S1	Rye	Powder	United States	2021	Not specified
S2	Rye	Powder	Korea	2021	Not specified
S3	Rye	Powder	Korea	2022	Not specified
S4	Rye	Powder	Canada	2021	Not specified
S5	Rye	Powder	Canada	2021	02/16/2024
S6	Rye	Powder	United States	2023	Not specified
S7	Rye	Powder	Canada	2023	09/18/2024
S8	Whole wheat	Powder	Australia and United States	2023	09/19/2024
S9	Whole wheat	Powder	United States	2023	05/01/2024
S10	Whole wheat	Powder	United States	2023	05/01/2025
S11	Whole wheat	Powder	United States	2023	07/04/2024
S12	Wheat	Powder	Australia	2023	09/20/2024
S13	Wheat	Powder	Korea	2023	08/29/2024
S14	Wheat	Powder	United States	2023	10/18/2024
S15	Wheat	Powder	Canada and United States	2023	09/20/2024
S16	Wheat	Powder	Australia and United States	2023	10/23/2024
S17	Wheat	Powder	Italy	2023	06/22/2024
S18	Wheat	Powder	France	2023	05/12/2024
S19	Wheat	Powder	France	2023	07/04/2024
S20	Wheat	Powder	France	2023	05/10/2024
S21	Wheat	Powder	France	2023	12/13/2023
S22	Wheat	Powder	United States	2023	05/05/2024
S23	Wheat	Powder	Canada and United States	2023	10/25/2024

[a]MM/DD/YYYY

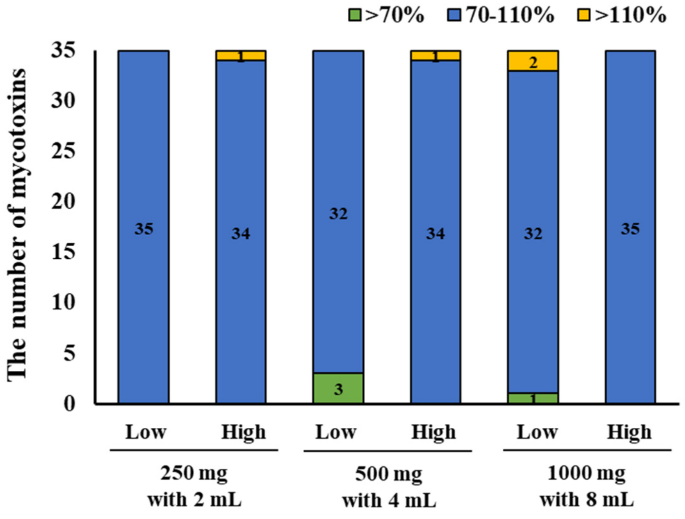

Figure S1. The recoveries of different sample weight and extraction solvent volume for LC-MS/MS at fortification level of low (5 μg/kg for AFs, OTs, FBs and EAs, 25 μg/kg for T-2, HT-2, DAS, 15-AS, Fus-X, DON, 3-AcDON, 15-AcDON, ZEA, ZEA-4S, α-ZOL and β-ZOL, 50 μg/kg for NIV) and high (4 times of low level).

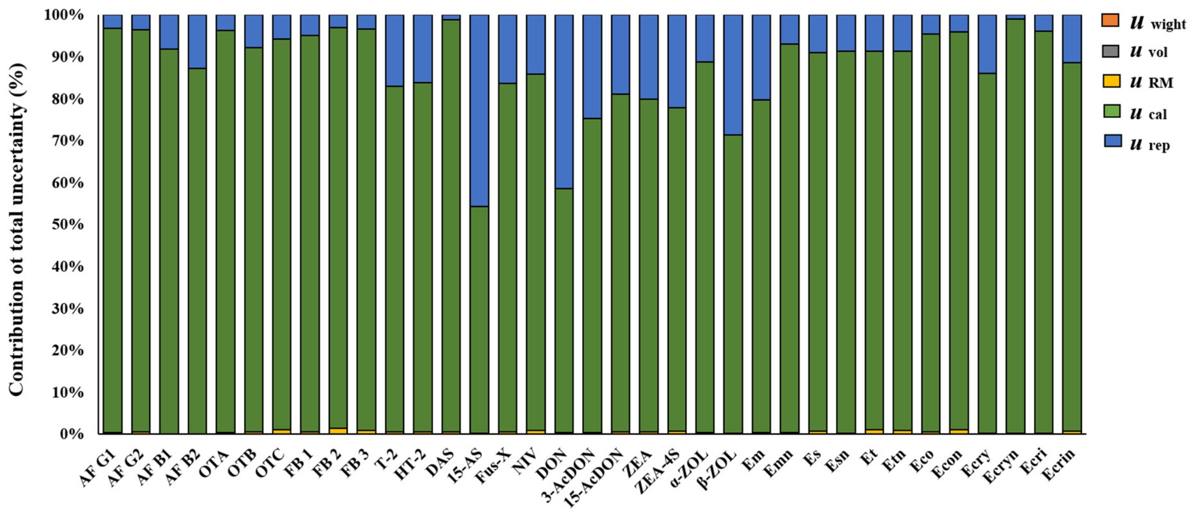

Figure S2. Contribution of each parameter of uncertainty in wheat (u_{weight}: sample weight, u_{vol}: extraction solvent volume, u_{RM}: reference material, u_{cal}: calibration curve, u_{rep}: repeatability)

Table S2. Recoveries and matrix effects of mycotoxins in the alternative clean-up sorbents (at low and high fortification level).

Analyte	Without Cleanup			Slica gel			Florisil			$C_{18}EC$			PSA			Al_2O_3			Z-sep		
	Recovery (%)		ME[a] (%)	Recovery (%)		ME (%)	Recovery (%)		ME (%)	Recovery (%)		ME (%)	Recovery (%)		ME (%)	Recovery (%)		ME (%)	Recovery (%)		ME (%)
	Low[b]	High[c]		Low	High		Low	High		Low	High		Low	High		Low	High		Low	High	
AF G1	99	94	-48	88	93	-50	81	83	-44	98	101	-49	88	82	-50	102	99	-46	74	76	-22
AF G2	98	96	-29	89	97	-23	84	84	-13	96	96	-22	88	73	-18	109	100	-13	71	72	-1
AF B1	74	90	-60	77	85	-62	52	44	-59	94	101	-64	83	70	-58	106	97	-55	74	88	-41
AF B2	101	98	-63	79	87	-60	48	46	-59	91	89	-64	91	82	-63	102	104	-61	75	85	-43
OTA	97	94	-73	54	66	-51	89	87	-45	91	88	-45	88	85	-45	91	71	-40	11	12	-22
OTB	78	83	-41	60	80	-23	103	82	-17	84	83	-8	103	87	-20	101	77	-11	13	13	11
OTC	96	97	-35	33	42	3	45	35	24	93	87	20	52	53	4	38	28	22	21	33	29
FB 1	98	94	7	67	77	11	75	78	15	93	89	35	26	29	0	15	19	30	19	19	38
FB 2	86	91	6	55	64	4	78	60	17	90	91	28	52	54	7	11	12	23	15	14	45
FB 3	80	82	8	52	69	4	71	57	13	87	85	17	33	40	-2	11	18	19	16	16	45
T-2	87	94	-47	75	95	-16	103	86	-7	86	88	-2	90	95	-12	107	87	-2	45	66	-27
HT-2	76	74	-29	77	103	-21	109	88	-9	88	84	-17	81	89	-21	106	97	-8	57	83	11
DAS	82	84	-8	78	100	10	110	93	23	95	93	15	75	73	14	105	92	28	52	76	38
15-AS	72	73	-23	74	99	-12	106	87	-5	73	72	-15	72	84	-13	95	96	-5	58	80	9
Fus-X	79	88	-44	78	100	-36	98	83	-21	99	93	-35	74	83	-27	100	100	-18	58	79	0
NIV	78	81	-80	70	91	-78	100	87	-72	91	90	-77	101	75	-67	86	90	-65	44	66	-35
DON	73	77	-29	130	104	-12	103	109	-17	92	90	-28	87	85	11	108	104	-7	75	80	24
3-AcDON	95	85	-82	70	99	-79	112	91	-77	107	91	-78	90	82	-77	99	101	-76	54	79	-47
15-AcDON	81	94	-80	84	101	-74	103	92	-74	94	96	-76	97	83	-75	98	100	-73	59	78	-45
ZEA	89	76	-67	49	85	-56	79	90	-53	91	95	-58	87	80	-55	83	76	-53	48	76	-22
ZEA-4S	75	70	-41	80	90	-51	104	98	-51	88	81	-62	94	89	-52	106	93	-49	50	79	-11
α-ZOL	74	75	-18	60	72	-15	78	75	-3	93	100	-4	92	90	-12	89	70	0	48	70	20

β-ZOL	76	76	-9	84	68	6	72	86	-1	78	81	-7	107	90	11	99	79	15	67	75	34
Em	76	75	-15	70	87	-22	65	58	-6	86	87	-18	89	71	-19	88	98	-11	51	62	15
Emn	78	74	-23	65	85	-18	57	46	-8	86	84	-15	103	86	-9	106	87	3	51	72	18
Es	73	100	-46	72	81	-31	52	45	-21	86	82	-30	101	83	-15	79	77	-9	55	77	22
Esn	80	76	-29	50	70	-25	31	32	-20	75	73	-13	77	80	-23	74	74	-12	46	64	28
Et	77	79	-24	53	65	-19	56	38	-6	85	82	-18	97	86	-11	70	73	-5	63	79	16
Etn	69	76	-26	44	59	-17	35	29	-9	70	75	-17	74	84	-14	64	63	-7	50	69	19
Eco	97	99	-23	47	61	-17	42	35	-6	95	88	-16	60	83	-4	67	65	-3	55	74	18
Econ	71	76	-22	49	65	-14	44	37	-2	85	92	-12	81	88	-6	73	74	-3	51	70	19
Ecry	74	85	-42	39	52	-35	33	28	-28	100	92	-28	47	28	-29	50	60	-27	52	73	8
Ecryn	88	82	-33	45	61	-23	37	32	-15	92	88	-46	51	43	-19	76	71	-20	42	61	-38
Ecri	72	73	-34	39	47	-11	34	27	-2	86	82	-10	61	55	-6	44	53	1	52	72	31
Ecrin	75	82	-49	41	52	-32	37	30	-29	86	84	-42	34	45	-37	50	61	-29	46	67	-3

[a]ME, Matrix effect (%). [b-c] Low level, 5 μg/kg for AFs, OTs, FBs and EAs, 25 μg/kg for T-2, HT-2, DAS, 15-AS, Fus-X, DON, 3-AcDON, 15-AcDON, ZEA, ZEA-4S, α-ZOL and β-ZOL, 50 μg/kg for NIV; High level, 4 times of low level.

Table S3. Linear range and linear equation of multiple mycotoxins and derivatives in cereals.

Analyte	Linear range (μg/kg)	Linear equation
AF G1	0.25-10	y= 20555.08×-1438.74
AF G2	0.25-10	y= 12873.88×+81.01
AF B1	0.25-10	y= 21056.36×+1411.98
AF B2	0.25-10	y= 7047.47×-890.61
OTA	0.25-10	y= 7230.20×-355.61
OTB	0.25-10	y= 24126.23×-1158.20
OTC	0.25-10	y= 22222.76×-967.27
FB 1	0.25-10	y= 5207.62×+601.90
FB 2	0.25-10	y= 9946.90×+82.85
FB 3	0.25-10	y= 6158.33×-315.55
T-2	1.25-50	y= 7992.35×-1286.61
HT-2	1.25-50	y= 3375.92×+972.98
DAS	1.25-50	y= 23665.76×-30289.71
15-AS	1.25-50	y= 5778.87×+882.17
Fus-X	1.25-50	y= 524.58×+404.26
NIV	2.5-100	y= 443.48×-202.27
DON	1.25-50	y= 1789.29×+8353.36
3-AcDON	1.25-50	y= 614.91×-474.75
15-AcDON	1.25-50	y= 756.91×+60.13
ZEA	1.25-50	y= 6149.50×-2181.19
ZEA-4S	1.25-50	y= 1034.15×-854.36
α-ZOL	1.25-50	y= 5234.29×-3411.17
β-ZOL	1.25-50	y= 7657.65×+5162.47
Em	0.25-10	y= 2614.96×+1560.41
Emn	0.25-10	y= 7020.09×+139.63
Es	0.25-10	y= 13353.46×+801.72
Esn	0.25-10	y= 2830.25×+48.87
Et	0.25-10	y= 14529.88×+5137.20
Etn	0.25-10	y= 43757.28×+2580.55
Eco	0.25-10	y= 17169.30×+6717.09
Econ	0.25-10	y= 29628.62×+2483.07
Ecry	0.25-10	y= 10744.00×+1654.66
Ecryn	0.25-10	y= 12811.92×+435.02
Ecri	0.25-10	y= 15847.37×+1385.40
Ecrin	0.25-10	y= 7812.00×+515.76

Table S4. Stability result of mycotoxins in wheat under different storage conditions (n=3).

Analyte	Day1				Day3								Day7							
					4℃				-20℃				4℃				-20℃			
	Low[a]		High[b]		Low		High		Low		High		Low		High		Low		High	
	Re[c] (%)	SD[d]	Re (%)	SD	Re (%)	SD	Re (%)	SD	Re (%)	SD	Re (%)	SD	Re (%)	SD	Re (%)	SD	Re (%)	SD	Re (%)	SD
AF G1	93.7	4.9	101.7	2.1	90.5	8.6	94.8	5.5	87.4	11.6	84.3	10.1	98.9	0.9	102.7	8.0	99.4	2.4	98.8	2.9
AF G2	89.0	6.4	90.3	1.8	89.3	3.8	88.5	5.1	97.7	9.8	86.8	13.5	104.0	4.5	105.5	10.6	93.4	5.4	96.8	2.8
AF B1	81.5	6.6	98.0	6.4	91.0	4.6	91.1	4.2	95.0	6.7	95.9	3.8	134.2	25.4	88.3	1.8	90.0	7.4	77.4	16.8
AF B2	96.1	1.7	92.4	4.0	84.9	6.1	96.4	3.0	86.5	4.1	104.7	6.7	114.5	18.2	91.1	36.6	94.6	16.4	95.7	12.8
OTA	92.8	4.3	88.4	2.5	93.1	3.3	96.0	1.3	99.3	9.0	94.3	4.7	108.6	6.3	108.5	9.5	101.9	6.1	101.0	5.4
OTB	86.9	1.5	86.1	0.9	91.3	1.9	98.2	6.3	84.4	6.6	88.0	1.0	89.6	4.6	94.9	0.3	90.3	4.1	94.3	0.5
OTC	94.9	3.8	85.4	1.6	90.0	6.9	98.6	5.1	96.1	8.7	88.1	0.5	99.4	20.5	91.4	10.0	93.1	5.3	94.4	5.7
FB 1	95.2	0.3	88.5	1.4	99.9	5.6	92.3	2.8	89.7	3.1	81.3	3.4	92.5	16.1	77.2	7.3	81.2	11.1	48.7	34.4
FB 2	88.5	1.4	90.2	1.1	101.4	0.8	100.6	4.5	93.6	1.7	92.8	1.2	120.3	14.5	110.1	11.5	91.9	3.3	97.4	30.3
FB 3	86.2	2.4	83.7	1.9	95.2	5.6	98.6	3.2	91.3	7.8	89.0	2.4	98.1	9.5	91.9	9.3	81.2	12.2	90.8	25.6
T-2	83.0	3.2	85.7	2.2	98.8	7.9	101.1	3.9	89.8	4.5	89.9	8.7	100.0	0.6	93.3	13.1	83.7	1.6	77.4	21.9
HT-2	83.2	1.4	81.3	1.9	81.5	7.3	81.8	8.4	93.5	6.0	92.9	8.9	106.2	14.3	86.8	11.3	82.4	3.3	87.1	24.3
DAS	92.9	2.5	90.5	1.8	98.9	6.2	97.9	9.6	80.6	1.9	95.6	5.5	102.5	8.2	105.6	31.4	93.0	15.3	95.3	30.4
15-AS	85.2	0.6	84.5	1.5	83.5	3.2	82.5	2.4	81.6	0.5	86.8	1.9	65.9	4.1	64.7	1.4	81.3	2.3	87.3	12.7
Fus-X	101.7	12.9	99.0	2.2	97.8	8.6	89.9	4.9	87.6	10.2	104.1	5.6	94.1	11.2	108.0	3.2	99.0	11.6	105.3	0.7
NIV	93.8	3.4	93.7	2.4	91.8	8.1	87.8	2.5	84.1	5.3	89.6	7.0	92.4	10.7	81.0	3.6	82.7	11.4	80.7	3.5
DON	95.0	7.7	92.8	2.1	99.2	9.2	87.4	6.6	87.5	7.6	98.9	8.2	91.2	10.2	87.4	3.9	92.1	10.2	86.2	25.1
3-AcDON	106.6	11.8	92.1	1.8	99.1	4.1	94.5	3.3	108.3	4.6	92.7	1.4	98.1	6.5	95.7	10.1	96.1	5.2	107.5	3.7
15-AcDON	96.4	2.2	97.3	2.3	94.2	6.9	96.0	3.3	89.1	5.6	97.6	7.4	92.0	9.0	96.6	7.4	95.6	2.0	97.3	6.9

ZEA	95.1	4.6	96.2	3.1	95.9	3.4	95.0	2.5	99.4	2.2	103.2	6.8	109.5	3.5	98.9	8.2	99.1	1.3	90.3	7.1
ZEA-4S	84.8	5.5	83.7	3.6	96.5	7.5	91.9	7.9	82.6	1.0	90.5	5.6	65.7	4.5	69.7	6.5	98.5	6.7	91.3	1.7
α-ZOL	95.7	3.1	99.7	5.5	99.4	1.7	93.7	3.7	110.0	2.2	99.9	3.8	83.4	17.8	84.1	2.4	88.7	4.8	99.2	3.2
β-ZOL	88.9	2.4	81.2	1.7	87.6	6.0	88.1	5.9	85.3	5.1	87.6	2.6	85.5	4.1	103.5	15.8	84.8	2.9	80.3	5.3
Em	88.4	3.9	89.2	0.7	87.7	3.7	85.8	5.6	81.3	5.1	83.4	6.5	100.6	11.0	86.0	5.1	91.2	9.3	96.3	5.4
Emn	85.9	0.8	84.0	1.7	86.8	2.2	85.9	4.2	80.1	1.2	84.7	4.5	70.8	16.5	76.0	4.7	79.4	0.1	75.7	3.4
Es	87.9	1.2	83.9	1.6	81.8	3.2	83.0	1.1	80.0	7.0	95.8	5.4	84.9	10.7	79.8	10.7	104.0	4.0	93.9	2.4
Esn	88.8	1.0	81.8	2.2	86.2	8.3	84.5	5.8	84.7	8.1	80.7	0.9	90.5	9.1	87.6	12.9	83.7	9.1	89.8	4.2
Et	85.5	1.9	83.4	0.7	97.6	3.4	102.5	4.8	102.1	4.3	94.6	2.7	86.9	2.6	85.5	12.0	81.6	3.4	99.3	8.2
Etn	88.0	6.1	86.6	3.2	83.9	4.2	93.5	2.3	88.9	2.9	82.7	5.2	81.9	2.8	97.2	7.3	88.5	5.6	100.1	6.8
Eco	99.0	5.6	93.8	1.7	109.9	6.5	101.2	9.7	84.4	6.8	94.4	10.3	102.9	15.1	94.6	11.2	93.0	4.1	89.1	6.8
Econ	86.8	3.5	93.8	1.5	87.2	5.2	99.6	3.8	80.5	6.7	91.6	3.9	78.3	5.2	79.8	5.4	89.1	4.1	85.4	3.5
Ecry	85.8	1.9	93.3	1.1	90.2	10.1	101.1	1.8	95.0	1.9	95.3	3.0	86.9	3.3	95.3	13.5	82.5	1.7	97.1	21.9
Ecryn	89.3	3.4	87.3	1.9	84.4	9.8	83.5	3.7	88.1	3.0	89.6	8.5	86.7	7.5	86.0	0.8	77.1	0.2	89.8	2.1
Ecri	83.4	2.1	88.7	0.7	86.4	4.5	92.2	5.6	88.7	5.3	97.4	9.4	113.0	5.3	94.8	3.5	101.0	0.7	105.3	4.4
Ecrin	86.0	2.4	83.8	4.3	95.0	6.0	90.3	5.2	99.2	1.0	96.1	4.6	76.5	3.4	78.4	5.8	75.7	18.5	82.8	4.4

[a-b] Low level, 5 μg/kg for AFs, OTs, FBs and EAs, 25 μg/kg for T-2, HT-2, DAS, 15-AS, Fus-X, DON, 3-AcDON, 15-AcDON, ZEA, ZEA-4S, α-ZOL and β-ZOL, 50 μg/kg for NIV; High level, 4 times of low level. [c-d] recovery and standard deviation.
